# Delayed Azithromycin Treatment Improves Recovery After Mouse Spinal Cord Injury

**DOI:** 10.3389/fncel.2019.00490

**Published:** 2019-11-06

**Authors:** Timothy J. Kopper, Katelyn E. McFarlane, William M. Bailey, Michael B. Orr, Bei Zhang, John C. Gensel

**Affiliations:** ^1^Spinal Cord and Brain Injury Research Center, Department of Physiology, University of Kentucky, Lexington, KY, United States; ^2^College of Public Health, Shaanxi University of Chinese Medicine, Xianyang, China

**Keywords:** anti-inflammatory, monocyte, microglia, M1, M2, alternatively-activated, neuroinflammation, neuroprotection

## Abstract

After spinal cord injury (SCI), macrophages infiltrate into the lesion and can adopt a wide spectrum of activation states. However, the pro-inflammatory, pathological macrophage activation state predominates and contributes to progressive neurodegeneration. Azithromycin (AZM), an FDA approved macrolide antibiotic, has been demonstrated to have immunomodulatory properties in a variety of inflammatory conditions. Indeed, we previously observed that post-SCI AZM treatment reduces pro-inflammatory macrophage activation. Further, a combined pre- and post-injury treatment paradigm improved functional recovery from SCI. Therefore, for the current study, we hypothesize that post-injury AZM treatment will improve recovery from SCI. To test this hypothesis, we examined the therapeutic potential of delayed AZM treatment on locomotor, sensory, and anatomical recovery. We administered AZM beginning 30-min, 3-h, or 24-h following contusion SCI in female mice, and then daily for 7 days. AZM administration beginning 30-min and 3-h post-injury improved locomotor recovery with increased stepping function relative to vehicle controls. Further, delaying treatment for 30-min after SCI significantly reduced lesion pathology. Initiating AZM treatment 24-h post-injury was not therapeutically effective. Regardless of the timing of the initial treatment, AZM did not statistically reduce the development of neuropathic pain (mechanical allodynia) nor increase neuron survival. Collectively, these results add to a growing body of evidence supporting AZM’s translational potential as a therapeutic agent for SCI and other neuroinflammatory conditions in which patients currently have very few options.

## Introduction

Spinal cord injury (SCI) induces a complex heterogeneous inflammatory response largely mediated by resident microglia and infiltrating monocyte-derived macrophages. While these cells are capable of adopting a wide spectrum of beneficial and detrimental functions, the acute SCI microenvironment promotes pro-inflammatory macrophage activation (Kigerl et al., 2009). Pro-inflammatory macrophages and microglia are widely believed to be major contributors to the continued neurodegeneration and tissue loss observed following the initial mechanical SCI. Targeting macrophage activation acutely is, therefore, a promising therapeutic approach to improve recovery. However, to date there are no FDA approved drugs to target these pathways after SCI.

Azithromycin (AZM) is a widely-prescribed, FDA-approved, antibiotic with a well-established safety record. AZM has significant anti-inflammatory and immunomodulatory actions across a wide array of disease states (Murphy et al., 2008, 2010; Feola et al., 2010; Ivetić Tkalcević et al., 2012; Banjanac et al., 2012; Nujić et al., 2012; Polancec et al., 2012; Vrančić et al., 2012; Zhang et al., 2015; Amantea et al., 2016b; Gensel et al., 2017; Osman et al., 2017; Varano et al., 2017; Barks et al., 2019). Specifically, AZM promotes anti-inflammatory activation by inhibiting macrophage STAT1 and NF-κB signaling pathways (Haydar et al., 2019). Emerging evidence supports the use of AZM as a treatment for neurological conditions including stroke, retinal ischemia, spinal muscular atrophy, and neonatal hypoxic–ischemic brain injury (Amantea et al., 2016a, b; Osman et al., 2017; Varano et al., 2017; Barks et al., 2019). Previously, we demonstrated that AZM improves tissue sparing and locomotor recovery in a mouse model of contusion SCI when dosing is initiated 3 days prior to injury (Zhang et al., 2015). In our previous work, the neuroprotective effects of AZM were coincident with increased anti-inflammatory and decreased pro-inflammatory macrophage activation (Zhang et al., 2015). More recently, we established that delaying treatment for 30 min post-injury substantially decreases markers associated with pro-inflammatory (M1) macrophage activation while significantly increasing anti-inflammatory (M2) macrophage markers (Gensel et al., 2017). Further, we recently found that AZM decreases neurotoxic, pro-inflammatory macrophage activation independent of its antibiotic properties (Zhang et al., 2019). Collectively, these findings highlight AZM as a promising SCI immunomodulatory therapeutic; however, the long-term effects of delayed AZM treatment are unknown. For the current study, we hypothesized that post-injury AZM treatment improves recovery from SCI. Specifically, we evaluated the therapeutic efficacy of post-SCI AZM treatment on long-term locomotor, sensory, and anatomical recovery. Initial treatment was delayed 30 min, 3-h, or 24 h post-injury and recovery evaluated for 4 weeks after injury.

## Materials and Methods

### Experimental Design

The current data includes the combined results of three independent studies.

#### Study One

Mice (*n* = 10/group) were treated with vehicle (initiated at 30 min post-injury) or AZM (160 mg/kg/day) by oral gavage beginning 30 min, 3 h, or 24 h after a moderate-severe (75 kdyn) T9 contusion SCI. Drug and vehicle administration was continued daily for 7 days post-injury (dpi). Locomotor function of all animals, as determined by the Basso Mouse Scale (BMS), was assessed prior to injury and again at 1, 3, 7, 14, 21 and 28 dpi. At 28 dpi, all the animals were sacrificed for the generation of spinal cord sections for histological analyses of tissue sparing, lesion length, and neuron survival. One mouse was euthanized due to surgical complications. Three mice were excluded based upon a prior exclusion criteria for abnormal impactor parameters reported by the Infinite Horizons (IH) SCI device (indicative of a bone hit or spinal cord movement during injury; *n* = 2) and abnormally high functioning locomotor behavior at 1 dpi (BMS >3) indicative of incomplete injury (*n* = 1).

#### Studies Two and Three

**Figure 1:**
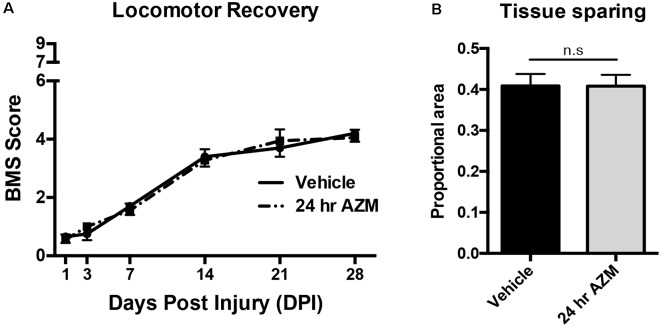
Azithromycin (AZM) administration beginning 24 h after injury is not therapeutically effective. Adult (4-month-old) female mice received a moderate-severe thoracic T9 contusion spinal cord injury (SCI; 75-kdyn). AZM was first administered at 24-h post-injury and then daily for 7 days (160 mg/kg/day). Functional recovery was assessed before injury and at 1, 3, 7, 14, 21, and 28 dpi. **(A,B)** Initiating AZM treatment 24-h after SCI did not improve locomotor recovery relative to vehicle control (*p* = 0.99, *n* = 9–10) or improve tissue sparing [as defined by glial fibrillary acidic protein (GFAP) reactivity] at the lesion epicenter at 28 dpi relative to vehicle (n.s. = not significant, *p* = 0.98, *n* = 7–8). Mean ± SEM.

Mice (*n* = 10/group/study) were injured and treated as study one except that the 24 h delayed treatment group was discontinued due to clear therapeutic ineffectiveness in study one (Figures 1A,B; *p* > 0.98 for all outcomes). In addition, horizontal ladder performance and measures of neuropathic pain (Von Frey, mechanical allodynia) were collected prior to injury and at 26 and 27 dpi, respectively. Group sizes for studies two and three (*n* = 10) were calculated based upon* a priori* power analysis of the BMS behavior data collected in study one. Specifically, we estimated that with a significance of α = 0.05, a power of 1-β = 0.80, and expected levels of animal attrition, that we would need an additional 20 animals per group. Two mice in study two and three mice in study three were excluded based upon* a priori* impact or behavioral criteria for incomplete/abnormal injuries.

Rostral-caudal neuronal survival was only assessed in studies two and three because unknown temperature inconsistencies during study one tissue processing and sectioning caused tissue folding and tissue loss, making histological analysis impossible. Discrepancies between *n*’s in behavioral vs. histological analyses are due to the fact that mice without an obvious and fully intact injury epicenter (due to tissue processing complications) were not included for histological analyses. Final animal numbers are reported in the figure legends and/or results.

### Animals

Experiments were performed using 4-month-old female C57BL/6 mice (Jackson Laboratory, Bar Harbor, ME, USA). Animals were housed in IVC cages with *ad libitum* access to food (Teklad Irradiated Global 18% Protein Rodent Diet) and purified water. Housing is set to maintain a 14 h light/10 h dark cycle, at 70°F and 50% humidity. All experimental procedures were conducted during the light cycle and were performed in accordance with the guidelines and protocols of the Office of Research Integrity and with approval of the Institutional Animal Care and Use Committee at the University of Kentucky.

### Spinal Cord Injury

Animals were anesthetized *via* intraperitoneal (i.p.) injections of ketamine (100 mg/kg) and xylazine (10 mg/kg). Following a T9 laminectomy, a moderate-severe thoracic SCI was produced using the IH injury device (75-kdyn; Precision Systems and Instrumentation; Scheff et al., 2004). Any animals receiving SCI with abnormalities in the force vs. time curve generated by the IH device were excluded from analysis. These abnormalities are indicative of bone hits or instability in the spinal cord at the time of injury and occurred <10% of the time. After injury, muscle and skin incisions were closed using monofilament suture. Post-surgery, animals received one subcutaneous injection of buprenorphine-SR (1 mg/kg) and one injection of antibiotic (5 mg/kg, enrofloxacin 2.27%: Norbrook Inc., Lenexa, KS, USA) in 2 ml of saline and were housed in warming cages overnight. Animals continued to receive antibiotic subcutaneously in 1 ml saline for 5 days. AZM (160 mg/kg) or vehicle (1% methylcellulose) was delivered in 0.1-ml volume *via* oral gavage daily beginning 30 min, 3 h or 24 h after injury and again daily for 7 days post-injury. Animal health and the incision site were monitored throughout the course of the study. Bladder expression was performed on injured mice twice daily.

### Behavioral Analysis

All experimental animals were assessed using the BMS to score hindlimb function as previously described (Basso et al., 2006). Mice were tested in an open field for 4 min before surgery and at 1, 3, 7, 14, 21, and 28 days post-injury (dpi). Each hindlimb was scored separately based on movement (e.g., ankle placement and stepping) and whole-body coordination and trunk stability were also scored; the average of both hindlimb scores was used to generate a single score for each animal. A score of 0 indicated complete paralysis and a score of 9 indicated normal locomotion. Animals receiving a score of 3 or higher at 1 dpi, or less than 2 at 28 dpi were excluded from the study based on* a priori* statistical assessment of over 450 prior 75 kdyn mouse SCI surgeries. These scores are rare (greater than 2 standard deviations from mean BMS score) and are indicative of surgical/injury abnormalities (bone hit, low/high impact force, etc.).

### Mechanical Allodynia Testing

Mechanical allodynia was measured using the manual up-down approach with von Frey monofilaments as described previously (Chaplan et al., 1994). Animals were first acclimated to the testing apparatus consisting of a wire mesh floor within an acrylic enclosure. A monofilament was pressed perpendicularly against the plantar surface of the hindlimb until bent, beginning with the 1.4 g monofilaments and ranging from the 0.4 g to 6.0 g monofilaments. Fifty percentage withdrawal threshold was calculated and reported as the average of both hind paws at each time point (Chaplan et al., 1994). Positive responses include rapid paw withdrawal, paw shaking and/or paw licking. Paw movement due to normal locomotor activity and responses occurring after the removal of the filament were not considered positive responses and were excluded from analysis.

### Tissue Processing and Immunohistochemistry

At 28 dpi, mice were anesthetized then transcardially perfused with cold PBS (0.1 M, pH 7.4), followed by perfusion with cold 4% paraformaldehyde (PFA). Dissected spinal cords (1 cm) were post-fixed for another 2 h in 4% PFA and subsequently rinsed and stored in cold phosphate buffer (0.2 M, pH 7.4) overnight at 4°C. On the following day, tissues were cryoprotected in 30% sucrose for 3 days at 4°C, followed by rapidly freezing and blocking in optimal cutting temperature (OCT) compound (Sakura Finetek USA Inc., Torrance, CA, USA) on dry ice. Tissue was systematically randomized into blocks (each block contained spinal cords from four subjects) with equal group distribution to ensure uniformity of staining across groups and blocked tissue was stored at −80°C before sectioning. Tissue blocks were cut in serial coronal sections (10 μm) and mounted onto Colorfrost plus slides (Fisher #12-550-17).

Spinal cord sections were stained with Eriochrome Cyanine (myelin) and anti-Neurofilament (1:1,000, Aves labs: NF-H) to visualize damage and thereby identify the epicenter and length of each lesion as described previously (Zhang et al., 2015). To examine the epicenter in greater detail, slides were stained with glial fibrillary acidic protein (GFAP; 1:500, Aves: GFAP) and macrophage marker F4/80 (1:1,500, AbD serotec, MCA497) primary antibodies overnight at 4°C, followed by Alexa Fluor 488 goat anti-chicken (1:1,000, Life Technologies A11039) and goat anti-rat Alexa Fluor 633 (1:1,000, Thermo Fisher Scientific: A-21094) secondary antibodies for 1 h at room temperature. To assess neuron survival, slides were subjected to antigen retrieval for 5 min in hot citrate buffer pH 6, incubated with rabbit anti-NeuN (1:4,000, Novus Biologicals NBP1-77686) primary antibody overnight at room temperature, then biotinylated goat anti-rabbit (1:500, Vector BA-1000) secondary antibody for 2 h at room temperature, then elite-ABC (prepared according to manufacturer’s instructions, Vector PK-6100) for 2 h at room temperature, and finally DAB (prepared according to manufacturer’s instructions, Vector SK-4100) with nickel additives for 5 min at room temperature. Slides were cover-slipped with Permount mounting medium (Fisher Scientific, Hampton, NH, USA) or Immu-Mount (Thermo Scientific, Waltham, MA, USA) for brightfield and fluorescent stains, respectively.

Images were taken using a C2+ laser scanning confocal microscope (Nikon Instruments Inc., Melville, NY, USA) or a ZEISS Axio Scan.Z1 (Munich, Germany), then analyzed using the MetaMorph analysis program (Molecular Devices, Sunnyvale, CA, USA) or HALO Image Analysis Platform (Indica Labs, Albuquerque, NM, USA). To identify the rostral-caudal lesion length, Eriochrome Cyanine and Neurofilament (EC/NF) stained tissue sections were examined at 100 μm intervals rostral and caudal from the lesion epicenter until damage became entirely localized to the dorsal column totaling less than 2% of the total spinal cord area. To quantify tissue sparing at the lesion epicenter (i.e., the tissue section with the least amount of intact EC/NF stained tissue) the regions of dense GFAP positive staining were outlined and measured using the MetaMorph analysis program (Molecular Devices, Sunnyvale, CA, USA) or HALO. These spared areas as defined by the GFAP+ glial scar closely align with areas of Neurofilament positive axons as determined previously (Zhang et al., 2015; Freria et al., 2017). Lesion area, intact tissue area, and overall spinal cord size were used to calculate the percentage of spared tissue at the lesion epicenter. While not quantified directly, F4/80 positive macrophages were used as a secondary tracing guide in any areas of ambiguity as they densely accumulate within the core of the lesion as described previously (Wang et al., 2015). To quantify neurons, NeuN-stained cells within the gray matter were quantified using HALO software. Spinal cross-sections at 100 μm intervals from the epicenter were analyzed, and average values were calculated from equidistant rostral/caudal sections. Folded or torn sections were excluded from analysis.

### Statistical Analysis

Investigators blinded to experimental conditions performed all data acquisition and analysis. Statistical analyses were completed using GraphPad Prism 6.0 (GraphPad Software). Data were analyzed using one- or two-way ANOVA followed by Dunnett’s or Holms–Sidak *post hoc* tests for multiple comparisons to the vehicle groups. Chi square and independent-sample *t*-tests were used when appropriate. Results were considered statistically significant at *p* ≤ 0.05. All data are presented as mean ± SEM unless otherwise noted. Figures were prepared using Adobe Photoshop CC 2014 (Adobe Systems) and Prism 6.0.

## Results

### Post-injury Administration of Azithromycin Improves Locomotor Recovery After SCI

**Figure 2:**
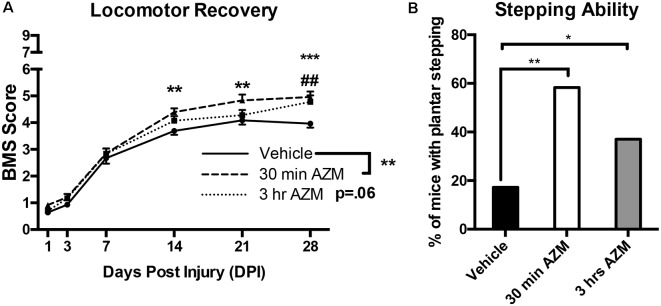
Early AZM administration improves locomotor recovery in SCI mice. Adult (4-month-old) female mice received a moderate-severe thoracic T9 contusion SCI (75-kdyn). AZM was first administered at 30-min or 3-h post-injury and then daily for 7 days (160 mg/kg/day). Functional recovery was assessed before injury and at 1, 3, 7, 14, 21, and 28 dpi. **(A)** Mice treated with AZM beginning 30-min post-injury displayed significantly improved locomotor recovery relative to vehicle (main effect of treatment vehicle vs. 30 min, *p* = 0.004) with significant improvement from 14 to 28 dpi (**,***p < 0.05 30 min vs. vehicle, Holms–Sidak’s *post hoc*). Mice first treated at 3 h post-injury had increased recovery relative to vehicle (*p* = 0.06 main effect of treatment vehicle vs. 3 h) with significant improvements at 28 dpi (^##^*p* < 0.05 vs. vehicle Holms–Sidak’s *post hoc*). **(B)** Mice treated at 30-min recovered significantly improved frequent plantar stepping frequency than vehicle controls (58% and 17% respectively, Chi-squared, *p* = 0.001). Similarly, mice treated at 3-h recovered significantly more frequent plantar stepping relative to vehicle (37% and 17%, respectively, Chi-squared, *p* = 0.04). *n* = 24–29, mean ± SEM. *p < 0.05.

As described previously, initiating AZM treatment 30 min after SCI, followed by repeated daily doses for 7 days, mediates an immunomodulatory shift in macrophage phenotype resulting in the downregulation of markers associated with pro-inflammatory M1 macrophage activation and upregulation of anti-inflammatory M2 markers (Gensel et al., 2017). Because this type of macrophage phenotypic transition is often associated with an increase in reparative functions after SCI, we sought to determine whether post-injury administration of AZM led to long-lasting locomotor improvement up to 28 days after injury. AZM was administered beginning 30 min, 3 h, or 24 h post-injury and then daily for 7 days in three compiled studies. The 24 h delayed treatment group was discontinued due to clear therapeutic ineffectiveness in study one (Figures 1A,B; *p* > 0.98 for all outcomes). The 30 min and 3 h administration groups, however, displayed improved locomotor recovery relative to vehicle. Specifically, overall locomotor recovery out to 28 dpi, as measured by the BMS, varied as a function of treatment (*p* = 0.008 main effect of treatment; time × treatment interaction *p* = 0.02). Locomotor recovery significantly improved when AZM was administered at 30-min relative to vehicle (*p* = 0.004 main effect; Figure 2A). This treatment effect was significant beginning at 14 dpi (*p* = 0.005; Figure 2A). Delaying the initial dose for 3-h after SCI improved recovery relative to vehicle (*p* = 0.058 vs. vehicle, main effect) with significant improvements by 28 dpi (*p* = 0.0004).

By 28 dpi, delaying AZM treatment by either 30 min or 3 h after SCI significantly improved locomotor function vs. vehicle with an average BMS score of ~5 for AZM groups vs. ~4 for the vehicle-treated group (Figure 2A). A transition from a score of 4–5 is largely dependent on the mouse’s ability to fully support its body weight on its hind legs while stepping. Because we observed this group-dependent separation along the 4/5 score of the BMS scale, we further quantified stepping ability. As seen in Figure 2B; a significantly greater proportion of mice treated with AZM beginning at either 30 min or 3 h post-injury recovered frequent plantar stepping function vs. vehicle controls (Chi-squared, *p* = 0.001 and *p* = 0.04, respectively). We also evaluated proprioceptive hindlimb stepping function with the horizontal ladder task. There were no differences among groups prior to SCI (*p* = 0.99, one-way ANOVA, data not shown). However, since few animals in the vehicle group recovered stepping function and were able to perform the horizontal ladder task, results of this test were not compared among groups after SCI.

### Azithromycin and Neuropathic Pain

**Figure 3:**
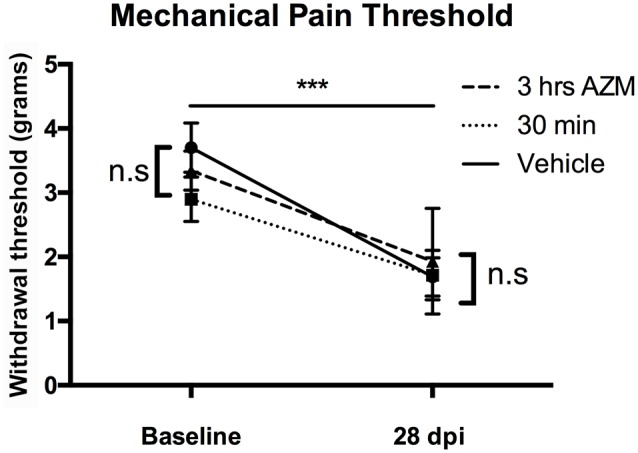
AZM administration does not alter the development of mechanical allodynia in mice after SCI. Gram-force withdrawal threshold to Von Frey filaments stimuli decreased after SCI, relative to baseline, indicative of the development of allodynia after SCI (***p > 0.0001). However, AZM did not alter the development of allodynia at 27 dpi relative to vehicle (n.s. = not significant, *p* = 0.89). There were no baseline differences in withdrawal threshold prior to injury (*p* = 0.29). *n* = 15–20, mean ± SEM.

We recently reported that AZM is an analgesic that alleviates chronic SCI pain when dosed 30 min prior to measuring heat-induced hyperalgesia (Gensel et al., 2019). Here, we expanded upon this by testing whether AZM limits the development of neuropathic pain (mechanical allodynia) after SCI. As seen in Figure 3, SCI induced allodynia (*p* < 0.0001), however, AZM administration beginning 30 min and 3 h following injury did not significantly reduce long-term pain responses (mechanical allodynia) relative to vehicle-treated animals at 27 dpi (*p* = 0.89).

### The Effect of Post-SCI Azithromycin Treatment on Tissue Pathology

**Figure 4:**
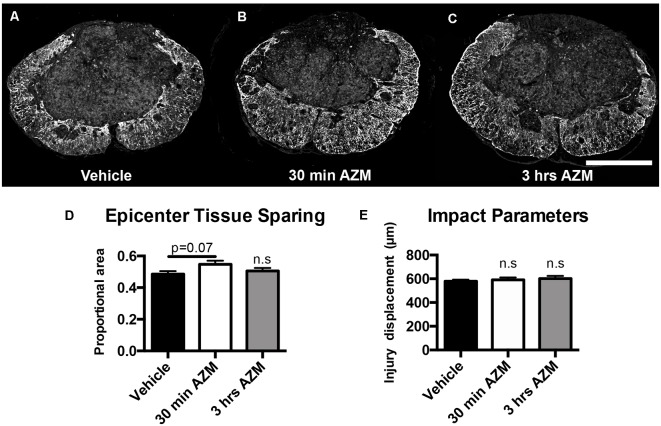
AZM does not significantly increase long-term, 28-day tissue sparing at lesion epicenter. Tissue sections representative of the mean values for **(A)** vehicle or AZM beginning **(B)** 30 min or **(C)** 3 h post-SCI. **(D)** Quantification of tissue sparing at 28 days post-injury based on GFAP reactivity did not demonstrate any statistically significant differences across groups, however, the mean tissue sparring was higher in the 30-min group (56%) than the vehicle-treated group (49%; *p* = 0.07). **(E)** The injury displacement values were equal across groups (*p* = 0.69) indicative of comparable injury severities prior to treatment administration. *n* = 19–25, mean ± SEM. Scale bar: 500 μM. n.s = not significant.

**Figure 5:**
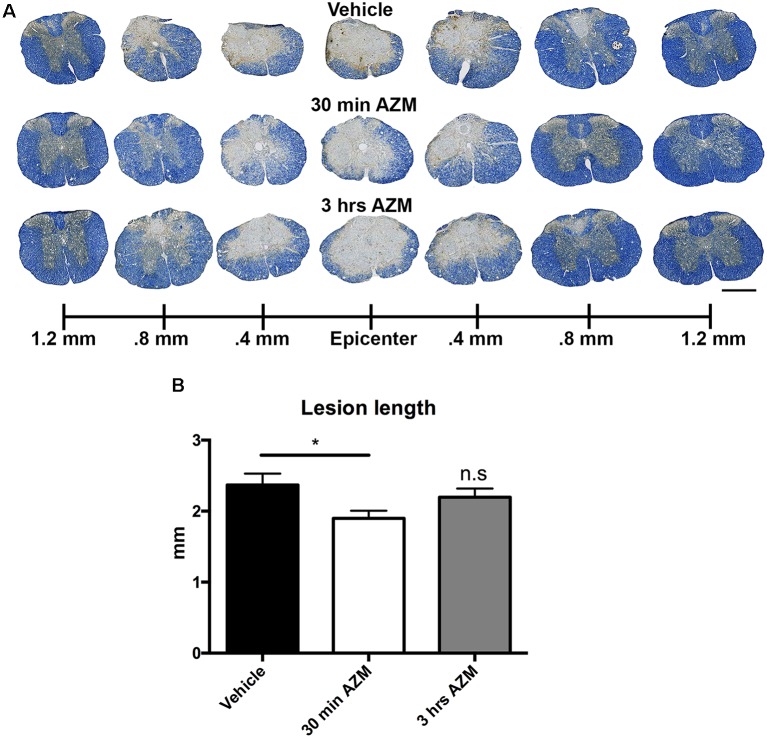
Post-SCI treatment with AZM reduces rostral-caudal lesion length. **(A)** Representative images of EC/NF stained sections 28 days after SCI detailing the lesion characteristics for vehicle, 30-min AZM, and 3-h AZM administration paradigms. **(B)** AZM administration beginning 30 min post-injury reduced overall rostral-caudal lesion length to an average of 1.9 mm relative to the 2.4 mm average in vehicle-treated animals (*p* = 0.03). This is evident in **(A)** by the relatively intact spinal cord at 0.8 mm rostral and caudal to the epicenter in the 30 min AZM vs. vehicle. group. Mice with treatment beginning at 3-h post-injury had a slightly lower average lesion length at 2.2 mm, however this was not statistically significant (*p* = 0.55). *n* = 23–29, mean ± SEM. Scale bar: 500 μM. * *p*< 0.05; n.s = not significant.

**Figure 6:**
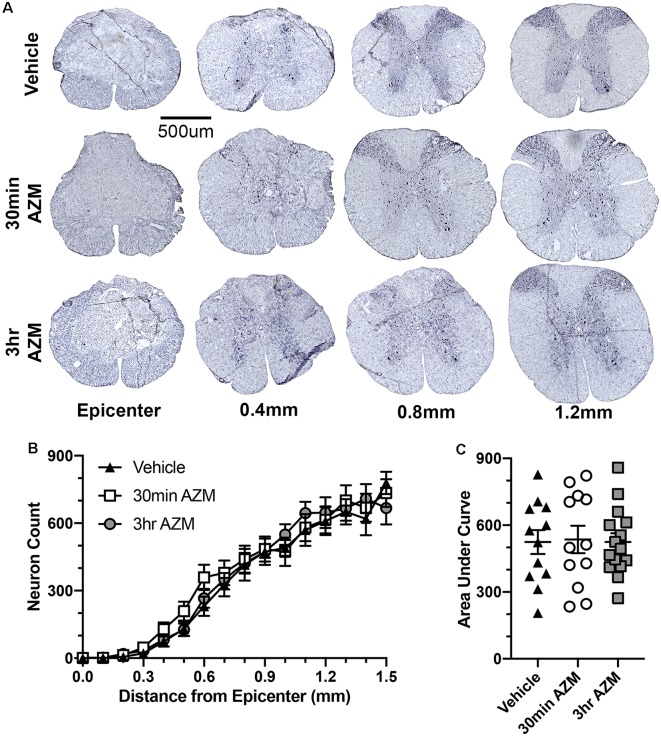
AZM treatment does not affect neuron sparing.** (A)** Representative examples of NeuN-stained spinal cross-sections ≤1.5 mm from the lesion epicenter 28 days after SCI. Treatment with AZM does not affect neuron sparing throughout the rostral-caudal extent of the lesion (ANOVA main effect of treatment *p* = 0.26; **B**) or the total neuron sparing according to area under the curve analysis (ANOVA *p* = 0.98; **C**). *n* = 14–18, mean ± SEM.

Previously, we observed improved locomotor recovery along with increased tissue preservation using a combined pre- and post-SCI dosing paradigm for AZM (Zhang et al., 2015). As seen in Figures 4A–E, the effect post-SCI AZM treatment on tissue sparing at the lesion epicenter did not reach statistical significance for either the 30 min or 3 h treatment groups in the current studies (*p* = 0.07 and 0.54, respectively). AZM administration significantly reduced the mean rostral-caudal lesion length when given 30 min after injury (*p* = 0.03; Figures 5A,B); the 3-h delivery timepoint was not statistically significant (*p* = 0.55; Figure 5). Given that pro-inflammatory macrophages are neurotoxic and that AZM limits this activation *in vitro* we sought to quantify whether post-SCI AZM treatment improved the number of surviving neurons at 28 dpi (Zhang et al., 2015, 2019). However, AZM treatment did not affect neuron sparing throughout the rostral-caudal extent of the lesion (ANOVA main effect of treatment *p* = 0.26; Figures 6A,B) or total neuron sparing according to area under the neuron by distance curve (ANOVA *p* = 0.98; Figure 6C).

## Discussion

The clinical use of methylprednisolone, the only FDA-approved drug to complete phase three clinical trials for SCI to date, has fallen substantially in recent years with concerns that its side effects may out-weigh its clinical benefits. As a result of this decline, many individuals with a SCI have been left with no pharmacological treatments for their injuries. Here, we provide evidence that azithromycin (AZM) treatment, initiated after SCI, improves recovery. Specifically, delaying treatment for 30 min or 3 h post-injury significantly increased locomotor recovery in mice. We observed therapeutic effects even when delayed for 3 h, although less robust than our 30-min timepoint. When delayed 24 h after injury AZM administration had no discernable therapeutic effect. Azithromycin is routinely given to SCI individuals with limited side effects (Evans et al., 2005, 2011) and our results here demonstrate that AZM may have a viable therapeutic window as a neuroprotective treatment for SCI.

Clinically, AZM is widely used for its antibiotic properties, however, increasing evidence indicates that the neuroprotective effects are a result of a distinct cellular mechanism. Specifically, AZM has a remarkable ability to accumulate within cells, in particular, within phagocytes such as macrophages (Zimmermann et al., 2018). Subsequently, AZM appears to inhibit the activation of the NF-κB signaling cascade, a potent regulator of macrophage activation states (Aghai et al., 2007; Cigana et al., 2007; Feola et al., 2010; Haydar et al., 2019). In support of the concept that the immunomodulatory effects of AZM are independent of its antibacterial actions, we recently developed a library of AZM derivatives with reduced antibiotic properties. Indeed, even compounds with reduced antibiotic activity retained the ability to blunt pro-inflammatory macrophage activation and macrophage-mediated toxic effects on neurons (Zhang et al., 2019). Similarly, investigators in other disease models have utilized AZM as an immunomodulatory agent and have attributed its efficacy to its anti-inflammatory effects on macrophage physiology (Feola et al., 2010; Kitsiouli et al., 2015; Amantea et al., 2016b). We have identified a similar mechanism of action for AZM in SCI previously (Zhang et al., 2015, 2019; Gensel et al., 2017). Specifically, we found that AZM administration 30 min post-injury at doses of 10, 40, or 160 mg/kg decreased pro-inflammatory M1 macrophage gene expression at 3 dpi while the lowest (10 mg/kg) and highest (160 mg/kg) doses increased anti-inflammatory M2 macrophage gene expression at 7 dpi (Gensel et al., 2017). One small caveat to this, and the current study, is that all of the mice receive prophylactic antibiotic (enrofloxacin) during surgical recovery. It is thereby possible that there is a drug interaction effect, although their intended antimicrobial mechanisms have distinct cellular targets. Collectively, increasing evidence demonstrates that AZM accumulates in macrophages and improves outcomes by driving a shift in macrophages from pro-inflammatory and pathological M1 phenotypes to more reparative, anti-inflammatory M2 phenotypes. These AZM mediated shifts in macrophage activation states are thereby likely to be important factors mediating the long-term therapeutic benefits detailed in the current study, however, we did not specifically evaluate the impact of treatment timing on macrophage phenotype.

Our observations are consistent with reports of AZM being neuroprotective in other neurological conditions through the promotion of anti-inflammatory macrophage polarization. For example, Amantea et al. (2016b) demonstrated that administration of AZM is neuroprotective in a transient middle cerebral artery occlusion model of stroke. Importantly, they attributed AZM’s protective effects to its ability to drive macrophages to an anti-inflammatory M2 phenotype. Similarly, Varano et al. (2017) utilized AZM to target CNS inflammation in a rat model of retinal ischemia/reperfusion injury (pathology associated with glaucoma, diabetic retinopathy, and anterior ischemic neuropathy; Zheng et al., 2007). In that study, a single dose of AZM (150 mg/kg, i.p.) given after 50 min of ischemia, increased retinal ganglion cells survival by reducing the excitotoxicity and propagation of the macrophage response (Varano et al., 2017). Together with our prior observation of AZM decreasing M1 activation and increasing M2 macrophage *in vivo* after SCI (Zhang et al., 2015; Gensel et al., 2017), these studies suggest that AZM could have important therapeutic implications across many neurological conditions. In particular, CNS disorders such as traumatic brain injury, Alzheimer’s disease, and multiple sclerosis are known to be heavily influenced by inflammatory pathways. However, the efficacy AZM in these models remains to be determined. In many CNS disorders there is the added complexity of having both bone marrow derived monocytes/macrophages and microglial derived macrophage populations. While emerging evidence suggests that microglia may be more neuroprotective than monocyte-derived macrophages, we do not yet know through which population AZM exerts its therapeutic effects (Greenhalgh et al., 2018; Bellver-Landete et al., 2019). Our prior work using a combined pre- and post-injury AZM administration paradigm demonstrated that both microglia (*in vivo*) and macrophages (*in vitro*) are affected by AZM (Zhang et al., 2015; Gensel et al., 2017). The relative contribution of microglia vs. monocyte-derived macrophages to the therapeutic effects of AZM observed in the current study, however, remains unclear. To address these uncertainties, we are currently developing small molecule labeling techniques to track AZM after administration and methods to separate and analyze microglia and monocyte populations individually after SCI.

One limitation of the current study is that only one AZM dosing paradigm (160 mg/kg/day) was tested. Using typical interspecies allometric scaling, this dose may translate to a high, but still clinically relevant dose in humans (Nair and Jacob, 2016). However, additional dose-response studies or alternative formulations may improve the neuroprotective potential or therapeutic window of AZM. Indeed, we previously observed that AZM retains its ability to modulate macrophage phenotype even at substantially lower doses (10 and 40 mg/kg) when administration begins 30 min post-SCI (Gensel et al., 2017). Similarly, using alternative dosing strategies, Amantea et al. (2016a) demonstrated that both intravenous and intraperitoneal administrations of AZM were neuroprotective in a rodent model of stroke. In the case of intraperitoneal administration, AZM remained neuroprotective after stroke at doses from 150 mg/kg down to as low as 1.5 mg/kg, suggesting that alternative administration routes may offer greater effectiveness with reduced doses (Amantea et al., 2016b). We have also begun modifying AZM structure through medicinal chemistry to produce derivatives that lack antibiotic activity yet maintain their immunomodulatory effects on macrophages (Zhang et al., 2019). Ongoing work seeks to identify a derivative with enhanced anti-inflammatory activity, which may allow for further reductions in dosages prior to clinical translation.

The therapeutic efficacy of AZM in the current study decreased with the timing of the first oral dose after SCI with the 30-min time point being the most effective, 3-h being moderately effective, and no efficacy when the first treatment was delayed 24 h. It may be possible to extend this treatment window. For example, here we administered AZM by oral gavage, whereas faster and more direct methods such as intravenous administration may allow for more robust effects and a more flexible dosing window. In the treatment of stroke, intraperitoneal administration of AZM was effective when administered out to 4.5 h post-injury suggesting that alternative dosing approaches may be able to extend AZM’s dosing window post-SCI (Amantea et al., 2016a, b). Collectively, while the data showed here demonstrate that early oral administration of AZM is therapeutically effective, there are ample opportunities to both improve its efficacy and minimize any associated risks.

In our current dosing paradigm, we dosed for 7 days after injury, yet our most robust behavioral improvements are seen from days 14–28 post-injury. This delayed response is consistent with observations in humans and may be due in part to AZM’s ability to accumulate within macrophages. Indeed, in human patients treated with AZM for 3 days, AZM accumulation in monocytes is still evident by 14 days with minimal depletion (Wildfeuer et al., 1996). Presumably, there is a similarly prolonged presence of AZM within macrophages after SCI contributing to the neuroprotective effects well into chronic time points. We observed a similar response in using a pre-treatment dosing strategy in that we only saw significant behavioral differences relative to vehicle starting at 14 dpi (Zhang et al., 2015). This suggests that AZM accumulation in macrophages after SCI is sufficient to facilitate long-term improvements. Therefore, methods to enhance AZM’s targeted delivery to macrophages, such as with liposome-based drug delivery systems may facilitate greater therapeutic efficacy.

In the weeks and months following SCI, animals develop neuropathic pain with both hyperalgesia (increased pain from a stimulus that normally provokes pain) and allodynia (pain due to a stimulus that does not normally provoke pain; Deuis et al., 2017). Previously we demonstrated that AZM has analgesic properties when given to these chronically injured animals 30 min prior to pain testing (Gensel et al., 2019). This is in contrast with the current study in which AZM had no analgesic effects. The two key differences are that in the current study we dosed with AZM for 7 days, stopping 3 weeks prior to pain testing and we evaluated pain using a mechanical withdrawal (allodynia) test instead of thermal-induced pain test (allodynia and hyperalgesia). It is possible that the analgesic properties of AZM are modality-specific and AZM could block the development of heat but not mechanically induced pain responses. The results of the current study, however, suggest that AZM has no effect on the overall development of mechanical allodynia after SCI, but maintains promise as a pain relief alternative to opioid and non-steroidal anti-inflammatory based therapeutics. Further, it has become increasingly important to include tests for the affective components of pain in animal models when identifying new drugs to treat SCI (Kramer et al., 2016). It is possible that future studies evaluating the effects of AZM treatment on affective pain may reveal additional benefits of treatment.

## Conclusion

In the current study, we administered AZM through oral gavage after SCI and detected modest, yet significant therapeutic benefits. It is possible that alternate dosages and routes of delivery could broaden AZM’s treatment window and increase its therapeutic effects. Despite these challenges, AZM holds great promise in the treatment of SCI and a broad variety of other inflammatory disorders. Fortunately, AZM’s excellent safety history, and wide availability at essentially all healthcare centers would greatly reduce barriers preventing rapid use after injury (Uzun et al., 2014; Trifirò et al., 2017). The results of the current study add to a growing body of evidence supporting AZM’s translational potential as a neuroprotective agent for SCI and other neuroinflammatory conditions in which patients currently have very few options.

## Data Availability Statement

The datasets generated for this study are available on request to the corresponding author.

## Ethics Statement

The animal study was reviewed and approved by the Institutional Animal Care and Use Committee at the University of Kentucky.

## Author Contributions

TK, KM, WB, and JG designed the research. TK, KM, WB, MO, and BZ performed the research. TK, KM, MO, and JG analyzed the data. TK and JG wrote the article. All authors read and approved the final manuscript.

## Conflict of Interest

The authors declare that the research was conducted in the absence of any commercial or financial relationships that could be construed as a potential conflict of interest.
